# Pharmacologic treatment with CPI-613 and PS48 decreases mitochondrial membrane potential and increases quantity of autolysosomes in porcine fibroblasts

**DOI:** 10.1038/s41598-019-45850-4

**Published:** 2019-07-01

**Authors:** Bethany R. Mordhorst, Karl C. Kerns, Martin Schauflinger, Michal Zigo, Stephanie L. Murphy, Renee M. Ross, Kevin D. Wells, Jonathan A. Green, Peter Sutovsky, Randall S. Prather

**Affiliations:** 1 Department of Animal Sciences, University of Missouri, Columbia, MO United States; 2 Electron Microscopy Core Facility, University of Missouri, Columbia, MO United States

**Keywords:** Energy metabolism, Macroautophagy

## Abstract

A metabolic phenomenon known as the Warburg effect has been characterized in certain cancerous cells, embryonic stem cells, and other rapidly proliferative cell types. Previously, our attempts to induce a Warburg-like state pharmaceutically via CPI-613 and PS48 treatment did augment metabolite production and gene expression; however, this treatment demonstrated a Reverse Warburg effect phenotype observed in cancer-associated stroma. In the current study, we inquired whether the mitochondria were affected by the aforementioned pharmaceutical treatment as observed in cancerous stromal fibroblasts. While the pharmaceutical agents decreased mitochondrial membrane potential in porcine fetal fibroblasts, the number and size of mitochondria were similar, as was the overall cell size. Moreover, the fibroblasts that were treated with CPI-613 and PS48 for a week had increased numbers of large autolysosome vesicles. This coincided with increased intensity of LysoTracker staining in treated cells as observed by flow cytometry. Treated fibroblasts thus may utilize changes in metabolism and autophagy to mitigate the damage of treatment with pharmaceutical agents. These findings shed light on how these pharmaceutical agents interact and how treated cells augment metabolism to sustain viability.

## Introduction

A hallmark of the Warburg effect (WE) is predominate use of glycolysis as opposed to use of the tricarboxylic acid (TCA) cycle for energy production. The latter is most commonly used by differentiated cells^1^. Use of this seemingly atypical metabolism is thought to be more favorable for the production of biomass in rapidly proliferating cells^2^. More recently, research from others has evidenced that cancer associated fibroblasts aid in the in the metastasis and growth of cancer, whereby the stroma is coerced to elicit a Warburg effect-like metabolism deemed the ‘Reverse Warburg effect’^3–6^. In a prior study, we selected the pharmaceutical agents CPI-613 and PS48 in an effort to induce a WE-like metabolism^7^. The lipoate analog known as CPI-613 [6,8-*bis*(*benzylthio*)*octanoic acid*: hereafter called CPI] is a mitochondrial disrupter via inhibition of the mitochondrial enzymes pyruvate dehydrogenase and α-ketoglutarate dehydrogenase^8,9^. The allosteric small molecule PS48 (5-(4-*Chloro*-*phenyl*)-3-*phenyl*-*pent*-2-*enoic acid*) was used in effort to promote glycolysis by stimulation of the PI3K pathway as it activates phosphoinositide-dependent protein kinase 1 (PDK1)^10–12^.

We found that CPI and PS48 treatment combination did not increase cellular proliferation or decrease cell viability, but did induce changes in expression of genes implicated in cancer metastasis. Furthermore, the treated fibroblasts increased the concentration of pyruvate and glutamine in spent media thereby exhibiting more of a reverse Warburg effect of cancer stromal cells^7^. In the present study, we inquired how this treatment impacted the mitochondria as some research evidences reduced mitochondrial mass or an absence of detectible mitochondria in cancer stromal cells^6,13^.

## Results

### Impact of fibroblast treatment on mitochondrial potential and organelle staining intensity

**Figure 1:**
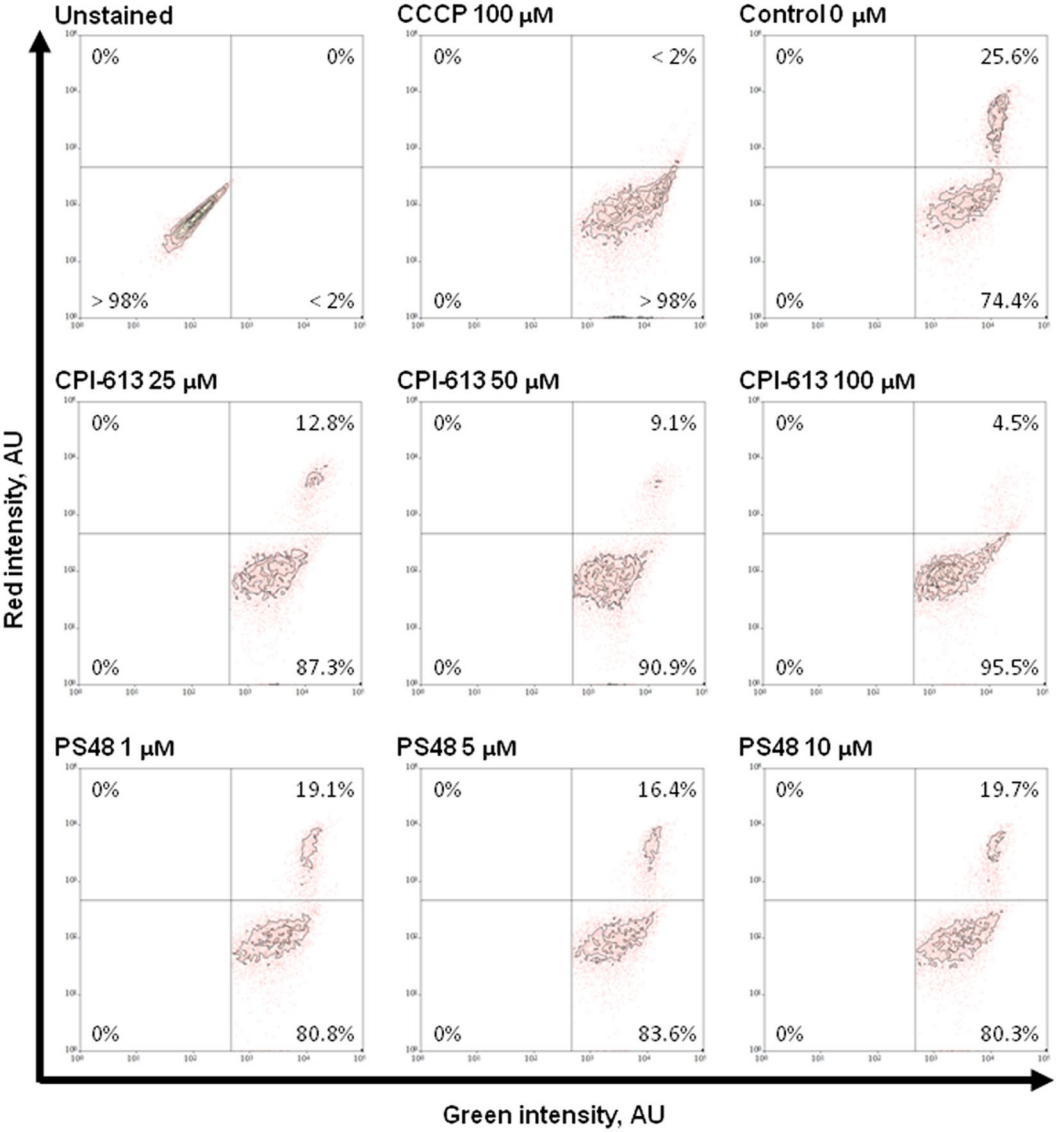
Flow cytometric scatter diagrams of CPI and PS48 dosage impacts on fibroblast mitochondrial membrane potential after treatment for 7 days.

Mitochondrial membrane potential (Δψ_m_) was measured using flow cytometric analysis of JC-10 staining in treated fibroblasts as an estimate of mitochondrial function for respiratory and tricarboxylic acid cycle capacity. Treatment with any concentration of CPI that was used (25, 50, or 100 µM) decreased Δψ_m_ (*P* < 0.01) by decreasing mean red intensity (≤503 AU vs. 950 AU) and increasing the proportion of cells in the low Δψ_m_ population (≥87.3 vs. 74.4%; Table 1). We found that treatment with 100 µM CPI yielded the highest proportion (*P* < 0.01) of fibroblasts with loss of Δψ_m_ (95.5% vs. ≤87.3% in lower CPI concentrations; Fig. 1; Table 1). Similarly, PS48 dosages increased the proportion of cells with Δψ_m_ loss (P = 0.04; ≥80.2% PS48 treatments vs. 74.4% in control [CON]; Fig. 1; Table 2), but did not significantly decrease mean red intensity (≥631 in PS48 treatments vs. 950 in CON; Table 2).

**Table 1:** Mitochondrial membrane potential function of fibroblasts treated with CPI-613 for 7 days.

Measure	Treatment^§^				SE	*P*-value
	0 µM	25 µM	50 µM	100 µM		
Red Intensity, AU	950^A^	503^B^	368^BC^	201^C^	87	<0.01
Green Intensity, AU	7808	6699	5865	5090	127	0.15
High Δψ_m_^‡^ population^ł˂^, %	25.6^A^	12.8^B^	9.1^B^	4.5^C^	2.3	<0.01
Low Δψ_m_ population^€˂^, %	74.4^A^	87.3^B^	90.9^B^	95.5^C^	2.3	<0.01

^§^Fibroblasts treated with 0, 50, or 100 µM CPI-613 by daily media changes at 24 ± 2 hours.

^‡^Δψ_m_ = Mitochondrial membrane potential.

^ł^Percentage of fibroblast population with high JC-10 red intensity and high membrane potential.

^€^Percentage of fibroblast population with low JC-10 red intensity and low membrane potential.

^ABC^ denotes differences between treatments within a row at a significance level of *P* < 0.05.

^˂^Corresponds to Fig. 1.

**Table 2:** Mitochondrial membrane potential function of fibroblasts treated with PS48 for 7 days.

Measure	Treatment^§^				SE	*P*-value
	0 µM	1 µM	5 µM	10 µM		
Red Intensity, AU	950	703	631	729	193	0.28
Green Intensity, AU	7809	7009	7361	6513	126	0.65
High Δψ_m_^‡^ population^ł˂^, %	25.6^A^	19.2^B^	16.4^B^	19.7^B^	3.2	0.04
Low Δψ_m_ population^€˂^, %	74.4^A^	80.8^B^	83.6^B^	80.3^B^	3.2	0.04

^§^Fibroblasts treated with 0, 5, or 10 µM PS48 by daily media changes at 24 ± 2 hours.

^‡^Δψ_m_ = Mitochondrial membrane potential.

^ł^Percentage of fibroblast population with high JC-10 red intensity and high membrane potential.

^€^Percentage of fibroblast population with low JC-10 red intensity and low membrane potential.

^ABC^ denotes differences between treatments within a row at a significance level of *P* < 0.05.

^˂^Corresponds to Fig. 1.

**Figure 2:**
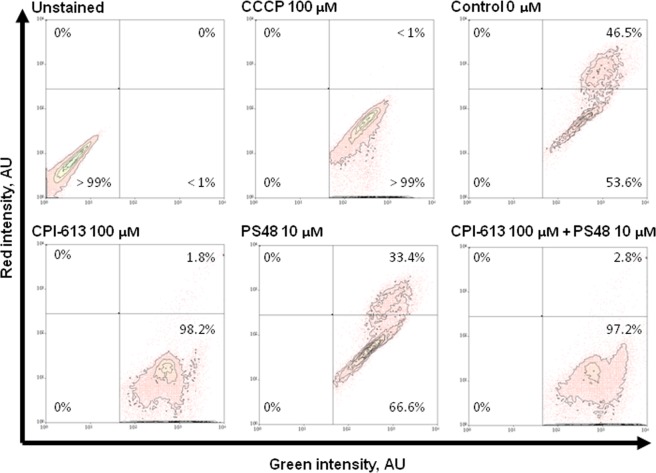
Flow cytometric scatter diagrams of mitochondrial membrane potential of fibroblasts treated for 7 days with a mixture of the two pharmaceutical agents (MIX; CPI 100 µM + PS48 10 µM), or without drugs (CON; 0 µM).

**Figure 3:**
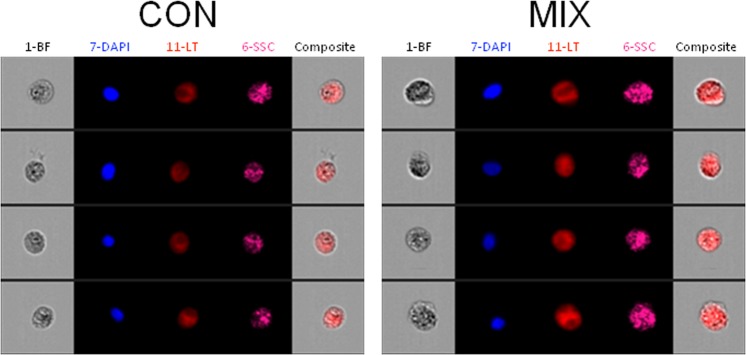
Image-based flow cytometric panels depicting representative fibroblasts treated for 7 days with a mixture of the two pharmaceutical agents (MIX; CPI 100 µM + PS48 10 µM), or without drugs (CON; 0 µM). Image panel: 1-BF = brightfield, 7-DAPI = nuclear staining, 11-LT = LysoTracker staining, 6-SSC = side scatter, Composite = Overlay of brightfield and LysoTracker images.

**Figure 4:**
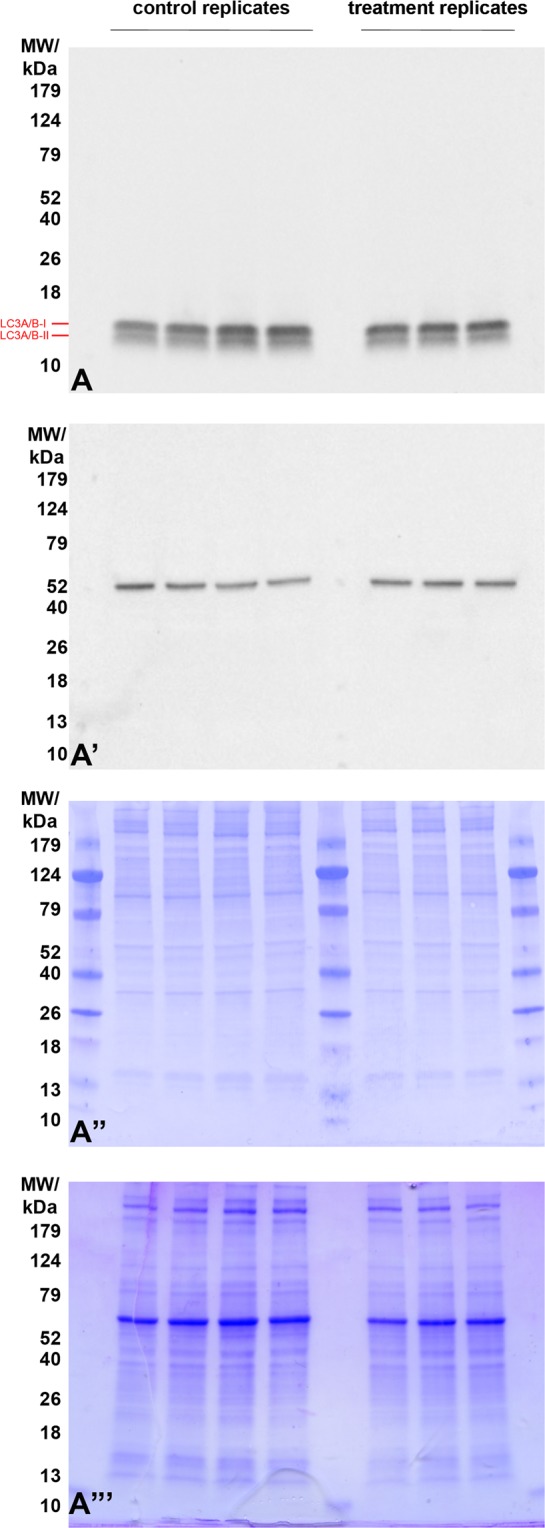
Western blot detection of Microtubule-associated proteins 1 A/1B light chain 3B (LC3A/B) in extracts from fibroblasts treated for 7 days with a mixture of the two pharmaceutical agents (MIX; CPI 100 µM + PS48 10 µM), or without drugs (CON; 0 µM). Red arrows highlight the expected molar weight of LC3 A/B proteins form I, and II (16 and 14 kDa, respectively). No difference was observed between treatment and control groups. (A’) Detection of β-tubulin shows comparable protein loads per lane. (A”) PVDF membranes stained with CBB after chemiluminescence detection shows comparable total protein loads per lane within each treatment, (A”’) residual gel after electrotransfer for protein load normalization purposes, and transfer efficiency control. Proteins were resolved on 8–20% gradient gel under reducing conditions, and 20 µg of protein was loaded per single lane.

Using the highest concentrations of PS48 (10 µM) and CPI (100 µM), we then investigated the impact of the drug mixture (MIX) on Δψ_m_ as well as mean intensity of MitoTracker green. Treatment with CPI or MIX decreased JC-10 mean red intensity (*P* < 0.01; ≤435.7 vs. ≥681.8 AU in CON and PS48), and the red/green intensity ratio (*P* < 0.01; ≤0.4 vs. ≥0.8 in CON and PS48; Table 3). The CPI or MIX treatments increased the percentage of fibroblasts with Δψ_m_ loss as compared to CON (*P* < 0.01; ≥97.2 vs. ≤53.6% in CON); whereas PS48 performed intermediately (*P* < 0.01; 66.6%; Fig. 2; Table 3). MitoTracker staining intensities were not impacted by treatments (Table 3). Increased intensity of LysoTracker staining in MIX cells indicated that there was an increased autolysosomal area within cells compared to CON (*P* = 0.04; 31,737 vs. 28,426 ± 402 AU; Table 4). The area of LysoTracker staining and brightfield area were measured via image-based flow cytometric analysis (Fig. 3). While LysoTracker area increased (*P* = 0.04) in MIX treated fibroblasts vs. CON (233 vs. 217 ± 2 AU), the brightfield area (cell size) remained the same (Table 4). Intensities of labeling with antibodies recognizing autophagic markers LC3 (Microtubule-associated proteins 1 A/1B light chain 3B) and BNIP3L (BCL2 [B-cell lymphoma 2] Interacting Protein 3 Like) were not significantly different between MIX and CON cells (Table 4). Western blot analysis confirmed that protein levels were not different amongst CON and MIX for LC3A/B-I (P = 0.77) or LC3A/B-II (P = 0.12) when normalized to α-tubulin (Fig. 4; red arrows highlight the expected molar weight of LC3A/B proteins).

**Table 3:** Mitochondrial membrane potential and staining intensity after 7 day pharmacological treatments in fibroblasts.

Measures	Treatment^§^				SE	*P*-value
	CON	CPI	MIX	PS48		
Red intensity, AU	924^A^	436^B^	435^B^	681^AB^	87	<0.01
Green intensity, AU	1144	1093	1132	903	127	0.52
Red/green ratio	0.81^A^	0.40^B^	0.39^B^	0.78^A^	0.06	<0.01
High Δψ_m_^‡^ population^ł˂^, %	46.5^B^	1.8^C^	2.8^C^	33.4^A^	3.3	<0.01
Low Δψ_m_ population^€˂^, %	53^A^	98.2^C^	97.2^C^	66.6^B^	3.3	<0.01
MitoTracker intensity, AU	817	838	767	699	73	0.55

^§^Fibroblasts treatments applied during daily media changes at 24 ± 2 hours; CON (0 µM), PS48 (10 µM PS48), CPI (100 µM CPI-613), or MIX (10 µM PS48 and 100 µM CPI-613).

^‡^Δψ_m_ = Mitochondrial membrane potential.

^ł^Percentage of fibroblast population with high JC-10 red intensity and high membrane potential.

^€^Percentage of fibroblast population with low JC-10 red intensity and low membrane potential.

^ABC^ denotes differences between treatments within a row at a significance level of *P* < 0.05.

^˂^Corresponds to Fig. 2.

^ł^Intensity of Mitotracker green FM staining.

**Table 4:** Image-based flow cytometric cellular features of fibroblasts treated for 7 days with MIX (100 µM CPI and 10 µM PS48) or CON (0 µM) during culture.

Measures	Treatment		SE	*P*-Value
	CON	MIX		
Brightfield cell area, AU^∑˂^	338	343	5	0.50
LysoTracker area, AU^‡˂^	217	233	2	0.04
LysoTracker intensity, AU^ł˂^	28,426	31,737	402	0.04
BNIP3L antibody intensity, AU^€^	4941	6562	1693	0.42
LC3 antibody intensity, AU^γ^	1899	2180	159	0.32

^∑^Area of cell in brightfield image from imaging cytometer.

^‡^Intensity of LysoTracker red staining.

^ł^Area of LysoTracker staining in image from imaging cytometer.

^€^Intensity of BNIP3L (BCL2 [B-cell lymphoma 2] Interacting Protein 3 Like) antibody staining.

^γ^Intensity of LC3 (Microtubule-associated proteins 1 A/1B light chain 3B) antibody staining.

˂Corresponds to Fig. 3.

### Impact of pharmacological treatment on fibroblast ultrastructure

**Figure 5:**
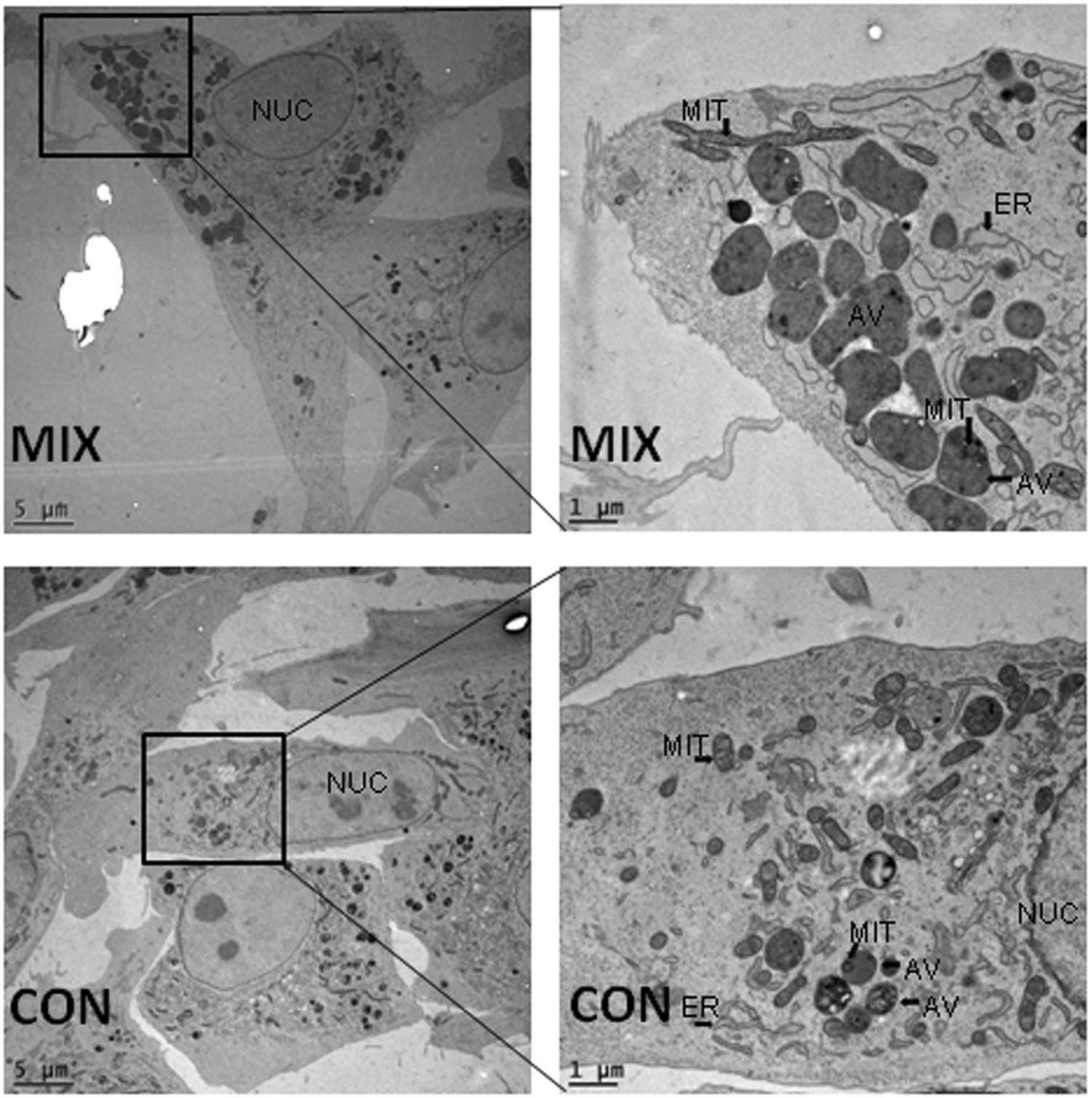
Electron micrographs of fibroblasts treated for 7 days with a mixture of the two pharmaceutical agents (MIX; CPI 100 µM + PS48 10 µM), or without drugs (CON; 0 µM). NUC = Nucleus, ER = Endoplasmic Reticulum, AV = Autophagic Vesicle, MIT = Mitochondria.

Electron microscopy was used to count and measure cellular features of interest. Area and perimeter of fibroblasts as well as their nuclei were not impacted by pharmaceutical treatment (see Table 5). The number, perimeter size, and area of mitochondria were not different between pharmaceutically treated and untreated fibroblasts (Table 6). Additionally, the proportion of the sum mitochondrial area (within a given cell) to the area of the cytoplasm (i.e. total cell area minus the area of the nucleus) was not significantly altered by treatment (Table 5). The number of autolysosomal vesicles more than doubled (18.4 vs. 7.8; Table 5) in the pharmaceutically treated fibroblasts compared to control. Some of these vesicles contained mitochondria that appeared to be undergoing degradation (Fig. 5).

**Table 5:** Ultrastructural cell features of fibroblasts treated for 7 days with MIX (100 µM CPI and 10 µM PS48) or CON (0 µM) during culture.

Ultrastructural measures	Treatment		SE	*P*-Value
	CON	MIX		
Cell perimeter size, µM	103.26	101.33	14.93	0.99
Cell area^§^, µM	380.02	362.78	122.91	0.52
Nucleus perimeter size, µM	28.18	29.43	4.19	0.53
Nucleus area^‡^, µM	54.88	56.19	13.87	0.84
Nucleus proportion of cell^ł^, %	16.92	17.08	1.63	0.95
Autolysosome number^€^, #	7.48	18.75	4.47	0.0038

^§^Area within cell perimeter minus the area of the nucleus.

^‡^Area within perimeter of nucleus.

^ł^Percent of cell area that was nucleus.

^€^Average number of autolysosomal vesicles within a cell.

**Table 6:** Mitochondrial parameters of fibroblasts treated for 7 days with MIX (100 µM CPI and 10 µM PS48) or CON (0 µM) during culture.

Ultrastructural measures	Treatment		SE	*P*-Value
	CON	MIX		
Total mitochondrial volume^§^, µM	5.52	6.13	2.22	0.61
Mitochondrial proportion of cell^‡^, %	2.32	2.36	0.83	0.91
Mitochondrial number^ł^, #	16.9	14.9	6.1	0.61
Mitochondrial perimeter size, µM	2.32	2.34	0.07	0.98
Mitochondrial area^€^, µM	0.22	0.25	0.01	0.23

^§^Sum area of all mitochondria within a cell.

^‡^Percent of total mitochondrial volume within cell area- nucleus area.

^ł^Average number of mitochondria within a cell.

^€^Average area within average mitochondrial perimeter.

## Discussion

We previously demonstrated that when PS48 and CPI are used in combination, gene expression and metabolite flux is augmented without changes in cellular proliferation^7^. Some of these cellular changes were also previously observed in cancer-associated stroma, which was deemed a “Reverse Warburg effect” metabolism. Our experimental goal was to determine if CPI and PS48 treatments previously used would impact mitochondrial quantities or capacity to function (membrane potential).

In the current study we found that mitochondrial membrane potential (Δψ_m_) of treated fibroblasts was lost (Figs 1, 2), which is indicative of mitochondrial oxidative phosphorylation impairment. Zachar, *et al*.^8^ demonstrated reduction of mitochondrial membrane polarization (as measured by JC-10) in response to CPI in a human lung carcinoma cell line (H460) treated with 240 µM CPI. They could decrease the proportion of cells with high mitochondrial polarization to 44% vs. 94% in controls after 3 hours of treatment with CPI without subsequent recovery (measured 3 hours post treatment). This study utilized porcine fetal fibroblast cells where CPI treatment for 7 days induced low Δψ_m_ in the majority of cells (>95% in both experiments). In our cell line, at least 53% of control cells already had lower Δψ_m_ prior to any treatment (Fig. 2; Table 3). All treatments in the current experiments did increase the number of low-functional mitochondria (Fig. 1; Tables 1, 2). It should be noted that while the same cell isolate was used in all experiments, dosage experiments were performed on a different cytometer at a different facility than in the experiment testing the drug combination, and this may account for the minor differences seen for Δψ_m_ measures between experiments 1 and 2 (Figs 1, 2; Tables 1, 2 vs. Table 3).

In our experiments, PS48 dosage correlated to an increased percentage of porcine fetal fibroblasts with low Δψ_m_ compared to control (Fig. 1; Table 2). Perhaps this decrease in mitochondrial function is in part due to increased glycolytic activity as in the prior study PS48 treated cells produced more pyruvate than control; however, we did not see differences in the production of either lactate or alanine^7^. Zhu, *et al*.^14^ identified PS48 as a small molecule that can be used to enhance reprogramming efficiency; the mechanism of which is attributed to the facilitation of glycolysis.

Inhibition of cellular respiration, or ‘reductive stress’, can elicit the production of reactive oxygen species (ROS) in mitochondria^15,16^. The MIX treatment likely results in chemical anoxia due to the compound CPI. Chemical anoxia can be induced by inhibition of ATP production which results in impaired mitochondrial function^15,17^. The pharmaceutical CPI disrupts mitochondrial metabolism via inhibition of the mitochondrial enzymes pyruvate dehydrogenase and α-ketoglutarate dehydrogenase^8,9^. Defective mitochondria are selectively degraded by autophagic encapsulation (a.k.a. mitophagy) and hydrolytic degradation via fusion with lysosomes [reviewed in^18^]. The fusion of autophagosomes to lysosomes has been referred to by several different terms [see^19^], but in this context we will use the term ‘autolysosome’. These were apparent as large, single-membrane dark structures in the cells (Fig. 5; labeled AV).

Pharmacological treatments increased the number of large autolysosomes to more than twice that of control fibroblasts (Table 5). Some autolysosomes contained mitochondria with partially intact membranes (Fig. 5; labeled MIT and AV). We did not quantify non-fused lysosomes, engulfing autophagic vesicles, or peroxisomes in electron micrographs due to uncertainty of differentiating between structures without further staining techniques. While LysoTracker intensity indicated increased area of lysosomes in MIX fibroblasts compared to CON, antibody detection of LC3 showed no differences between treatments. Tanida, *et al*.^20^ indicate that cellular level of LC3 may not be a good indicator of autophagy as lysosomal hydrolases degrade LC3 after the formation of autolysosomes; therefore resulting in “very low” LC3 content in autolysosomes [process reviewed in^21^]. Tanida, *et al*.^20^ concluded that at least for starvation-induced autophagy, lysosomal turnover seems to be a better indicator of autophagy.

We speculate ‘bulk autophagy’ may be induced due to chemical anoxia and serves as an adaptive route to self-preservation and survival for cells treated with a combination of CPI and PS48. We found no differences in BNIP3L (BCL2 and adenovirus E1B 19-kDa-interacting protein 3- like; also known as NIX) antibody labeling intensity detected amongst CON and MIX fibroblasts. In mammalian cells, mitophagy is in part mediated through BNIP3L; therefore, we propose that our treatment induces non-specific bulk autophagy in fibroblasts as opposed to selective removal of defective mitochondria.

In our micrograph sections, we found no differences in the size (perimeter and area) or number of mitochondria in pharmacologically treated fibroblasts compared to untreated controls (Table 6). Furthermore, there were no differences in MitoTracker Green FM intensity amongst treatments. It was not anticipated that mitochondria quantities would be similar between MIX and CON treatments given the impact of pharmaceuticals on Δψ_m_ loss and in doubling the number of autolysomes compared to untreated fibroblasts. One possible explanation is that treated fibroblasts have upregulated mitochondrial biogenesis to compensate for mitophagy of pharmaceutically-damaged mitochondria; however, we did not find statistical difference of expression in any of the known master genes of mitochondrial biogenesis^7^.

In a study from Meyer, *et al*.^22^ CPI was been used in combination with other reagents to block both glycolytic and citric acid cycle enzymes. To our knowledge, CPI and PS48 have not been used outside of our laboratory as a treatment combination to promote a WE-type metabolic phenotype. Contrary to our WE hypothesis, this pharmaceutical intervention seems to parallel more of a ‘Reverse Warburg effect’ (RWE) phenotype as there is more than twice the production of pyruvate^7^ and autolysomal vesicles compared to control fibroblasts. While we would not recommend this induction system to model cancer cell stroma, as there are notable biological differences, some of the results were similar to findings of protocols used to induce the RWE in cancer stroma. A number of studies demonstrate that cancer-associated fibroblasts exhibit autophagy/mitophagy, senescence, and predominant use of glycolysis^13,23–29^. Many have evidenced the role of oxidative stress or hypoxia in the induction of the RWE phenotype^3,4,24,27,30,31^. Others have revealed that a WE-like metabolism can be induced via nutrient deprivation (or starvation) in a HeLa^32^ and a human fibroblast cell line^33^. We speculate that our MIX-treated fibroblasts in this study experienced degrees of both oxidative stress (via chemical anoxia) and nutrient deprivation likely due to the action of the pharmaceutical agent CPI.

Here we demonstrate that fibroblasts treated with CPI and PS48 to induce a WE-like metabolism do not have altered numbers of mitochondria, though mitochondrial function is impaired. These fibroblasts appear to compensate and maintain viability without hindered proliferation through increased autophagy and augmented metabolism. The present study is novel in its approach to combine pharmaceutical reagents used in cancer and stem cell biology to promote metabolic shift. From this research, further insight as to how these reagents impact cellular health as well as alternative uses can be gleaned.

## Materials and Methods

All materials and supplies were purchased from Sigma-Aldrich, St. Louis, MO unless otherwise specified.

### Compliance with Ethical Standards

This article does not contain any studies with human participants performed by any of the authors. All procedures involving animals were approved by the University of Missouri Institutional Animal Care and Use Committee at the University of Missouri in Columbia, MO and were performed in accordance with these ethical standards.

### Fetal-derived fibroblast cell culture

Methods of cell line creation, culture, and pharmacologic treatments have been previously published^7^. Cell lines were derived from the dorsal portion of day 35 porcine fetuses which were frozen in liquid nitrogen (0.5 mL aliquots; ~80 cryogenic vials); fibroblasts such as these are available from the National Swine Research and Resource Center (http://nsrrc.missouri.edu/). A separate vial of fibroblasts was thawed for each replicate of each experiment; the same cell line was used throughout all experiments as well as those in prior research^7^. Incubators were maintained at 38.5 °C with a humidified atmosphere of 5% oxygen, 5% carbon dioxide, 90% nitrogen throughout all experiments. Cells were thawed and cultured in DMEM (1 g/L glucose, glutamine, and pyruvate with phenol red; Sigma, St. Louis, MO) supplemented with 15% FBS (Corning, Manassas, VA, USA) for seven days in T25 flasks (Corning, Corning, NY) with or without the addition of respective treatment concentrations of CPI-613 (CPI, Sigma, St. Louis, MO) or PS48 (Stemgent, Cambridge, MA). Media was changed daily in all flasks, i.e. those which received PS48 or CPI had new drugs applied daily (24 ± 2 hours).

In experiment 1, porcine fetal fibroblasts were treated with either CPI (25, 50, or 100 µM), PS48 (1, 5, or 10 µM), or as controls (0 µM) for 7 days of culture. In experiment 1, both CPI and PS48 stock aliquots were diluted to 10 mM in DMSO. In experiment 2, porcine fetal fibroblasts were treated with PS48 (10 µM), CPI (100 µM), a mixture of PS48 and CPI (10 µM and 100 µM), or treated as controls (0 µM) for 7 days of culture. In experiment 2, CPI stocks were diluted to 100 mM and PS48 stocks were 10 mM to eliminate potential confounding of DMSO quantity between the two pharmaceutical treatments (CPI and PS48). Cells were initially plated at 1 × 10^5^ cells/mL in T25 flasks. After 72 and 120 hours, cells were passaged to new T25 flasks. At 120 hours, cells in all treatments were plated (density: 5 × 10^5^ cells) to achieve ~80% confluence at 168 hours. At passage, cells were briefly rinsed with PBS containing 0.01 M EDTA and dissociated by brief incubation (37 °C) from flasks using 1 × Tryp- LE Express (Gibco, Denmark). At passage, cell number across treatments was determined and cells were re-plated with respective treatment media. Log transformations were made to data where appropriate prior to statistical analysis in order to achieve normal distributions. Trypan blue exclusion proliferation data was analyzed for effects of treatment, day, and the interaction of treatment by day by using the generalized linear model procedure of SAS 9.4. Differences with a *P*- value of <0.05 were considered significant. Values reported are least squared means with the highest standard error amongst the treatments.

### All flow cytometric data analyses

In all flow cytometry experiments, three replicates were collected for analysis and three technical samples for each treatment were run in the flow cytometer for each of the biological replicates. Data were assessed for normality via Univariate procedure in SAS 9.4 (SAS, Cary NC) which included the following normality tests: Shapiro- Wilk, Kolmogorov- Smirnov, Anderson-Darling, and Cramér-von Mises. Based on these tests, log and square root transformations were made where appropriate to achieve normality prior to statistical analysis. Data was analyzed by using the MIXED procedure of SAS 9.4 for main effect of treatment. Differences with a *P*- value of <0.05 were considered significant. Values reported are least squared means with the highest standard error amongst the treatments for flow cytometry acquisitions.

### Conventional flow cytometry data acquisition

For all conventional cytometry data collected, gating protocols were applied for analysis of a population free of debris and doublet cells by using plots of side scatter height × forward scatter height, forward scatter height × forward scatter area, and forward scatter height × forward scatter width.

### JC-10 mitochondrial membrane potential

The biphasic cationic dye JC-10 is able to infiltrate both the cytoplasm and mitochondria in a monomeric green emission form (525 nm); however, when mitochondrial membrane potential is elevated, JC-10 dye forms J-aggregates which have an orange emission (590 nm). This property allowed us to determine proportions of cells which contained mitochondria in a state of higher or lower mitochondrial membrane potential. Initially we tested CPI and PS48 dosages, and in a later experiment we measured a combination of the highest drug concentrations. Fibroblasts were dissociated and incubated at 37 °C for 30 minutes with 830 nM JC-10 (optimized by titration). In experiment 1, mitochondrial membrane potential was measured for cells stained with JC-10 by using a Beckman Coulter MoFlo XDP (Fullerton, CA) flow cytometer equipped with a 488 nm laser with 529/28 (FL1) and 580/23 (FL 2) dichroic filters using Summit 5.3 software (Beckman Coulter, Inc, Fullerton, CA). In experiment 2 JC-10 staining was measured by using a Beckman Coulter CyAN ADP Analyzer cytometer (Fullerton, CA) also equipped with a 488 nm laser with 530/40 (FL 1) and 575/25 (FL 2) dichroic filters and Summit 4.4 software (Beckman Coulter, Inc, Fullerton, CA). Channel compensation was adjusted for every replicate in experiments by measuring cells incubated with the mitochondrial membrane potential disrupter carbonyl cyanide 3-chlorophenylhydrazone (CCCP; 100 µM) for 2 hours prior to analysis as positive controls for monomeric JC-10 staining. For both experiments, a florescence intensity (FL 2 × FL 1) plot was divided into a four-square grid to determine mean intensities and percentages for cell populations. The horizontal axis was set based on an unstained control population and the vertical axis based on CCCP treated population (100 µM for 2 hours at 37 °C). Population percentages within quadrants as well as mean FL 1and FL 2 intensities were data collected for each population for every treatment.

### MitoTracker green cytometry acquisition

Fibroblasts were dissociated and incubated at 37 °C with 100 nM MitoTracker Green FM for 30 minutes. Mean fluorescence intensity in the FL1 (530/40 dichroic filter) channel was measured in MitoTracker stained fibroblast cells by using a Beckman Coulter CyAN ADP Analyzer cytometer (Beckman Coulter, Inc, Fullerton, CA). A plot of arbitrary event ‘count’ × FL1 intensity was used to determine mean intensity for each of the treatment replicates acquired. Unstained controls were used to determine positive florescence intensity in the FL1 intensity plot.

### Image-based flow cytometry data acquisition

Image-based flow cytometry (IBFC) data acquisition was performed as previously described, using a FlowSight flow cytometer (FS) fitted with a 20x microscope objective (numerical aperture of 0.9) with an imaging rate up to 2000 events/sec^34^. The sheath fluid was PBS (without Ca^2+^ or Mg^2+^). The flow-core diameter and speed was 10 μm and 66 mm/sec, respectively. Raw image data were acquired using INSPIRE® software. To produce the highest resolution, the camera setting was at 0.5 μm × 0.5μm pixel of the charged-coupled device. In INSPIRE® FS data acquisition software, the following channels were collected: two brightfield (channels 1 & 9), one LC3 or BNIP3L-Tritc (channel 3), one side scatter (SSC; channel 6), one Hoechst 33342 (channel 7), and one LysoTracker (channel 11), with a minimum of 10,000 cells collected. The following lasers and power settings were used: 405 nm (to excite Hoechst): 10 mW; 561 nm (to excite Tritc): 50 mW, 642 nm (to excite LysoTracker): 10 mW; and 785 nM SSC laser: 10 mW. Using IDEAS software, an image compensation matrix was calculated and applied to raw image files. The gating approach included cells in focus (histogram brightfield gradient RMS) and single cells (as opposed to doublet and debris; plot Hoechst area vs Hoechst aspect ratio).

### Lysotracker red, LC3 and BNIP3L antibody cytometry acquisition

A protocol similar to that used by^35^ was used for LC3 and BNIP3L staining preparation. Fibroblasts were dissociated, pelleted, and fixed in 4% paraformaldehyde then washed three times in cold PBS. Cells were then permeabilized in 100% cold methanol for 10 minutes at −20 °C and again washed in cold PBS three times. Afterward, cells were blocked for 30 minutes with 5% normal goat serum (NGS) at room temperature. Next, cells were incubated with a 1:250 dilution of either LC3 or BNIP3L primary antibodies overnight at 4 °C in 1% NGS PBS. The following day, cells were washed 3 times with 1% NGS PBS then incubated with 1:500 dilution of goat anti-rabbit Tritc and 1:1000 dilution of Hoechst and 100 nM LysoTracker red for 30 minutes at 37 °C.

### Western blot analysis

At least three replicates of fibroblast cells were cultured and treated for 7 days as CON or MIX as listed above. Cells were pelleted, washed, and resuspended in cold RIPA buffer (50 mM TRIS∙HCl, pH = 7.4, 150 mM NaCl, % (v/v) TrX-100, 1% (w/v) sodium deoxycholate, 0.1% (w/v) SDS, 1 mM DTT, 2 mM EDTA) then lysed via sonification on ice (50% amplitude for 2 seconds; Branson Ultrasonics) with Halt™ Protease and Phosphatase inhibitor (Thermo Fisher Scientific Cat # 78443). Proteins were resolved on 8–20% gradient gel (Bio-Rad, Hercules, CA, USA) under reducing conditions as described by Laemmli^36^, and 20 µg of the total protein (estimated by Bradford assay^37^) was loaded per single lane. Proteins were transferred to polyvinylidene difluoride membrane as described previously by Zigo, *et al*.^38^, blocked with 10% non-fat milk in TRIS-buffered saline (TBS, 50 mM TRIS∙HCl, pH = 7.4, 150 mM NaCl) supplied with 0.1% Tween 20 (TBST) and probed with anti- microtubule-associated proteins 1 A/1B light chain 3B (LC3A/B) antibody (clone D3U4C, cat# 12741; 1:1000; Cell Signaling Technology, Danvers, MA, USA). Afterwards, the membrane was stripped and re-probed with anti-β-tubulin antibody (clone E7; 1:4000; Antibody Registry ID: AB_2315513 from Developmental Studies Hybridoma Bank at the University of Iowa, Iowa City, IA, USA). Antibodies were diluted, as noted previously, in TBST supplied with 5% non-fat milk, and left to incubate overnight. Membranes were incubated with the appropriate species-specific secondary antibody such as, the HRP-conjugated goat anti-rabbit (GAR-IgG-HRP) or anti-mouse (GAM-IgG-HRP) antibodies (1:10,000 dilution; Invitrogen). The membranes were reacted with chemiluminescent substrate (Luminata Crescendo Western HRP Substrate; Millipore Sigma) and blots were screened with ChemiDoc Touch Imaging System (Bio-Rad, Hercules, CA, USA).Band intensities were quantified using ImageJ^39^ and LC3 band intensities were normalized to β-tubulin intensity and data were compared using the same statistical analysis listed above for cytometry.

### High pressure freezing and processing for electron microscopy

For ultrastructural analyses, treated (MIX; CPI 100 µM + PS48 10 µM) and untreated (CON; 0 µM) fibroblasts were treated daily and grown for 5 days. Afterward, fibroblasts were dissociated and plated in 6 well plates on gold-coated sapphire discs (3 mm in diameter; Wohlwend GmbH, Switzerland) and treated for additional two days. On day 7 of treatments, the cells were cryo-immobilized by high-pressure freezing by using the Wohlwend HPF Compact 02 and freeze substituted in acetone containing 0.1% (w/v) uranyl acetate, 0.5% (w/v) osmium tetroxide, and 0.5% (w/v) imidazole. Freeze substitution was performed in a Leica AFS by raising the temperature from −90 °C to 0 °C over a period of 18 h. Samples were infiltrated with Epon and polymerized at 60 °C. Thin sections (75 nm) were prepared, mounted on formvar/carbon coated copper grids, and imaged with the JEOL 1400 transmission electron microscope equipped with a CCD camera at an acceleration voltage of 80 kV. Images were exported in.dm3 format so that scale measurements would remain the same when opened in Image J software and no conversion between pixels to microns would be necessary. Cell features including all mitochondria and large autolysomes were outlined manually using the ‘freehand selection’ shape and measured using ImageJ^39^. For each of the three biological replicates, at least 5 cells were measured as technical replicates; in total 19 CON and 17 MIX cells were analyzed. Model fitting and testing were performed by the MIXED procedure in SAS (version 9.4) and analyzed for the main effect of treatment. Differences with a *P*-value of <0.05 were considered significant. Values reported are least squared means with the highest standard error amongst the treatments.

## Data Availability

All data are available upon request.
